# An Exhibition of Important Anatomical Books

**Published:** 1958-10

**Authors:** A. E. S. Roberts

**Affiliations:** Medical Library, University of Bristol


### AN EXHIBITION OF IMPORTANT ANATOMICAL BOOKS

A. E. S. ROBERTS

*Medical Library, University of Bristol*

"It is not necessary for the understanding of any science that we should be acquainted with its history", said Matthew Baillie in 1785. "There is, however", he went on to say, "a strong curiosity prompting us to inquire into the history of discoveries in any branch of science. . ."

This quotation comes from *Lectures and Observations on Medicine* by the late Matthew Baillie, printed in 1825 for private distribution among his friends according to directions left in a codicil to his will. The first part of his book consists of two introductory lectures to the course on anatomy given in Great Windmill Street in which he gives a very good outline history of anatomy.

In the Medical Library of the University of Bristol there are many books by authors who contributed something to the development of human anatomy, and a few of the important ones are at present on display in the Library.

Herophilus (300 B.C.) is said by Galen to be the first person publicly to dissect both human and animal bodies, and although his works are lost, Galen tells us that he recognized the brain as the central organ of the nervous system. We owe to him the first description of the lacteals. He was the first to grasp the nature of nerves other than those of the special senses. He divided nerves into motor and sensory, but continued to use the word neuron also for sinews and ligaments. He described the meninges and the torcular Herophili, which is named after him. He distinguished the cerebrum and the cerebellum, the fourth ventricle and, above all, the calamus scriptorius, which he named. The terms prostate and duodenum are derived from those which he used. He made the first clear distinction between arteries and veins.

Erasistratus, a younger contemporary of Herophilus, extended the observations of Herophilus on the lacteals. He also regarded the heart as the source of both arteries and veins and came very near to discovering the circulation of the blood. He realized the existence of the capillary system.

Galen (A.D. 130–200), in his voluminous works, gathered together all the medical knowledge of his time. His own knowledge of anatomy is best seen in his *De Anatomicis Administrationibus*, a translation of which is on display. His works dominated medicine until the time of Vesalius, thirteen centuries later.

It was not until the fourteenth century that dissections were generally legalised, so that in the time of Leonardo da Vinci (1452–1519) dissection of the human body was a well-recognized feature of medical instruction. Yet those early Renaissance anatomists made no progress, in spite of their opportunities for direct observation, for the reason that the lecturer not only remained aloof from the cadaver, but slavishly followed the written text; the actual dissector was inefficient and the process was necessarily a hurried one since there was no means of preserving the bodies from putrefaction.

The *Fasciculus medicinae* compiled by Johannes de Ketham in 1491 is the first printed medical book and consists of a collection of short medical treatises. In the library there is a facsimile of the 1493 edition which contains the *Anathoma* by Mundinus, the first book devoted solely to anatomy. This is on display open at a page illustrating an anatomy lecture. After the invention of printing the *Anathoma* was issued in several editions so that, having been originally written in 1316, it was in use altogether for well over 200 years.

Leonardo da Vinci in his anatomical drawings represented the structures of the human body with an accuracy and skill such as had never been seen before. A comparison of the typical fifteenth century *situs* figure in the *Fasciculus medicinae*

with Leonardo's *situs* figure (figures 3 and 4 in McCurrich, *Leonardo da Vinci, the anatomist*) shows how far Leonardo was ahead of his time in the matter of anatomical illustration.

The next book displayed is the most fully illustrated of the pre-Vasalian anatomies, *De dissectione partium corporis humani* by Charles Estienne, part of which was in preparation as early as 1530 though it was not published until 1545. The text is largely dependent on Galen. The chief defect of Estienne's work is the distorted ugliness of his figures, but they are the earliest, except for those of Leonardo, in which whole systems, venous and nervous, are shown.

The last of the pre-Vasalian illustrated anatomies is that of J. B. Canano (1515–1579), *Musculorum humani corporis picturata dissectio*, circa 1541. This pamphlet, a facsimile of which is displayed, was unique for its time in exhibiting each muscle in a separate figure and in approximately correct relation with the bones. Unfortunately the beautifully drawn figures, owing to the poor paper, have greatly deteriorated since his day.

In his superbly printed *De fabrica corporis humani*, Vesalius (1514–1564), having cast off the shackles of Galen, confined himself to what could be seen in the body. Not the least of the distinctions of his great work (a copy of the first edition 1543 is shown) is the dramatic and living impression that he succeeded in imparting to his figures which were so admirably executed by Jan Calcar, a pupil of Titian. Garrison and Morton consider the publication of this book was the greatest event in medical history since Galen: it ranks second only to Harvey's *De motu cordis* in importance.

The work of Bartolommeo Eustachius (1520–1574) *Tabulae anatomicae* was completed in 1552 but this fine collection of plates remained unprinted and forgotten in the Vatican Library till 1714 when they were published by Lancisi. Charles Singer is of the opinion that had these plates, which are more accurate than the work of Vesalius, been published in 1552, Eustachius would have ranked with Vesalius as one of the founders of modern anatomy.

A 1722 edition of his *Tabulae anatomicae* is in the Library, but it is too large to put on display. The same applies to *Tabulae anatomicae LXXIIX* (1632) by Guilio Casserio (1552–1616) and *Anatomia humani corporis* (1685) by Govert Bidloo, both of which contain magnificent copper-plate engravings.

Asellius (1581–1626) first distinguished the lacteals, at the beginning of the seventeenth century, to be a particular system of vessels, yet he was mistaken about their termination: for he believed that they ended in the liver, the trunk of the absorbents being still unknown. Attention to this subject was now, however, awakened among anatomists, and the error of Asellius was soon corrected by Pecquet (1622–1674). It remained still, however, to make the same progress in the human subject and Veslingius (1598–1649) is said to have observed in 1634 the lacteal vessels in man, and in 1649 the thoracic duct, and the lacteals were soon traced to their termination in it. The lacteals, however, form only a small part of the absorbent system and Rudbeck (1630–1702) has the honour of discovering the lymphatic vessels in 1650 while still a student at Padua, but he did not make the discovery public till 1653, a few months after Bartholin. Although the lymphatic vessels were now certainly discovered, yet anatomists were entirely ignorant of their use, and their relation to the absorbent system. This further important discovery was reserved for William Hewson (1739–1774).

The following books illustrate the history of the absorbent system: Gaspare Aselli, *De lactibus sive lacteis venis, in* Spigelius—Opera omnia, 1645 (too large to display). Aselli's original work, published in 1627 includes the first anatomical plates printed in colours, but the library unfortunately does not possess this. The works of Pecquet, Bartholin and Rudbeck, are contained in a collection by S. Hemsterhuys entitled *Messis aurea exhibens: anatomica: novissima et utilissima experimenta* 1659. This little book is on display.

William Hewson's *Experimental inquiries. Part II. Containing a description of the*

*lymphatic system* . . . 1774 is on display and the library also has the other two parts of his "Experimental inquiries" making in its complete form a rare and valuable possession.

The seventeenth century, owing to the invention of the microscope, produced some brilliant microscopical investigators; also the discovery of the process of making permanent anatomical preparations by means of injections opened up a new field of interest among anatomists. The study of anatomy had reached such proportions that it was now necessary to consider the preparation of the object under examination and following that the art of injection. But for injection to be satisfactory it was necessary to discover both the most suitable material and the means of adequately making the injection. We have Swammerdam (1637–1680) to thank for discovering the most suitable material and De Graaf (1641–1673) for inventing the syringe. A copy of De Graaf's *Opera omnia*, 1677, is on display, open at the illustration of his syringe which accompanies his work *De usu siphonis in anatomia*.

Marcello Malpighi (1628–1694) was one of the most brilliant microscopists of the seventeenth century. His most important discovery was capillary circulation which he described in 1661 in the lungs of a frog. It was announced in *De pulmonibus observationibus anatomicae* which consisted of two letters to Borelli, the second of which contains his discovery. It is republished in his *Opera omnia*, Vol. 2, p. 331, (which is on display), and an English translation by J. Young appeared in Proceedings of the Royal Society of Medicine, 1929–30, Section of the History of Medicine, vol. 23, pp. 1–11.

The anatomists of this period took immense pride in their preparations and paid more attention to their beauty than to their instructiveness. Paramount in this respect was Frederick Ruysch (1638–1731) a volume of whose *Thesaurus anatomicus* is exhibited. His name is connected anatomically with several discoveries, among which is a description of the valves of the lymphatics. The exact composition of the material used for injection by Ruysch, and also by many other microscopists, was kept secret.

The seventeenth and eighteenth centuries abound in originators of eponymous anatomical terms, and it is impossible in a short article to name all the anatomists who are perpetuated in medical terminology. Nor is there room to display all the relevant books possessed by the library. Among them, however, mention might be made of Spigelius (1578–1624) who has given his name to the Spigelian lobe of the liver; Francis Glisson (1597–1677) who was the first to give an accurate description of the liver and described the so-called Glisson's capsule; Thomas Willis (1622–1675) who in his *Cerebre anatome* gave the most exact account of the nervous system that had yet appeared. The Circle of Willis is still called after him.

Other names which might be mentioned are Stephen Blankaart (1650–1702) who succeeded for the first time in 1675 in injecting the capillary vessels, by which he obtained permanent and objective proof of the connection between the arteries and the veins; Giovanni Battista Morgagni (1682–1771) who founded modern morbid anatomy by his work *De sedibus et causis morborum*, in which he attempted to bring the symptoms of disease observed during life into relation with the phenomena revealed at autopsy; Jean Baptiste Senac (1693–1770) who wrote a valuable work on the structure of the heart which is said to have laid the foundation of cardiac pathology; William Cheselden (1688–1752) applied himself especially to osteology as did James Douglas (1675–1742); the elder Alexander Monro of Edinburgh founded the Medical School there; Albrecht von Haller (1708–1777) who was a prolific writer on anatomical subjects and whose textbook of physiology was used for many years; he was also an authority on the history of medicine; and John Hunter (1728–1793) who was one of the greatest surgical investigators the world had seen and who was eminent in every department he touched.

During this time anatomy consisted of a collection of descriptions of the different organs and groups of organs. But Xavier Bichat (1771–1804) in his *Anatomie générale appliqué à la physiologie et à la médecine* called general anatomy into existence when he distinguished the tissues common to all or peculiar to each part of the body. He thus changed the whole outlook of anatomy and physiology. His *Anatomie générale* is one of medicine's most important books.

### REFERENCES

Baillie, Matthew. *Lectures and observations on medicine.* London, 1825.
Duckworth, W. L. H. *Some notes on Galen's anatomy.* Cambridge, 1949.
McCurrich, J. P. *Leonardo da Vinci, the anatomist.* Washington, 1930.
De Lint, J. G. *Atlas of the history of medicine. I. Anatomy.* London, 1926.
Singer, C. *The evolution of anatomy.* London, 1925.
Dobson, J. F. "Erasistratus" *Proc. Roy. Soc. Med.*, 1927, **20,** (Sect. Hist. Med.), 21–28.
Dobson, J. F. "Herophilus of Alexandria". *Proc. Roy. Soc. Med.*, 1925, **18,** (Sect. Hist. Med.), 19–32.
Morton, L. T. *Garrison and Morton's Medical bibliography 2nd. ed.* London 1954.

### BOOKS ON DISPLAY

Galen. *On anatomical procedures.* London 1956.
"*Fasciculus medicinae*" (containing Mundinus *Anathoma* 1493). Facsimile. Florence 1925.
McCurrich, J. P. *Leonardo da Vinci, the anatomist.* Washington 1930.
Estienne, C. *De dissectione partium corporis humani.* Paris 1545.
Canano, J. B. *Musculorum humani corporis picturata dissectio.* ?1541. Facsimile. Florence 1925.
Vesalius, A. *De fabrica corporis humani.* Basel 1543.
Hemsterhuys, S. *Messis aurea exhibens; anatomica; novissima et utilissima experimenta.* Heidelberg 1659. (containing works of Pecquet, Bartholin and Rudbeck).
Hewson, W. *Experimental inquiries.* Part II. *Containing a description of the lymphatic system.* London 1774.
De Graaf, R. *Opera omnia.* Leyden 1677.
Malpighi, M. *Opera omnia.* Vol. 2. Littleburg 1687.
Ruysch, F. *Thesaurus anatomicus.* Amsterdam 1701–.
Glisson, F. *Anatomia hepatis* 1659.
Willis, T. *Cerebre anatomi* 1664.
Bichat, M. F. X. *Anatomie générale appliqué à la physiologie et à la médecine.* Paris 1801, Vol. 1.

